# Application and Uses of the Rubber Dam

**Published:** 1889-05

**Authors:** H. T. Lynch


ARTICLE VII.

### APPLICATION AND USES OF THE RUBBER DAM.

BY DR. H. T. LYNCH.

That all dental operations should be performed with a view toward accomplishing the best results and the most perfect operation, we presume no dentist will deny.

While we feel we should take into consideration the comfort of the patient, both during and after the operation, we should not allow our actions to be governed by his temporary inconvenience to such an extent that it will in any way effect the final results.

For instance, in filling teeth where pain is inflicted, we should not be controlled by the pleadings of the patient for temporary relief, to the detriment of his future benefit, and the durability of the operation. So, too, in the use of napkins, bibulous paper, spunk and the rubber dam: to all of which the patient will object, only thinking of his temporary comfort. However much the patient may object, no dentist will deny the necessity for keeping the cavity dry during the operation of filling the teeth. If, then, it be considered necessary to exclude the moisture in such operations, and to insert our fillings when the cavity is dry—or as nearly so as it is possible to make it—the next question arises, how is this result best accomplished? Is it by the use of bibulous paper, napkins, etc., or the rubber dam? That the rubber dam is superior to all other appliances for the purpose used; we believe all dentists will agree.

We frequently hear dentists speak of inserting large gold fillings by the use of napkins or bibulous paper, giving as an excuse that they can work faster, and that the patient objects to the use of the dam. Admitting the patient does object to the use of the latter, does the operator not know that it is impossible to keep the cavity dry during prolonged operations in any other way, and that he is only governed by his sympathy, and yielding to the wish of the patient for relief from a little pain or inconvenience, rather than guided by a desire to accomplish good results?

Do not all dentists know that the moisture from the breath, alone, is sufficient to destroy the cohesive qualities of gold, and thereby render the operation imperfect? To illustrate this, let any one stand ten or twelve inches distant from a window-pane, when a few blows from his oral cavity, on its surface, will show how much moisture may be deposited even at that distance. And there is certainly much more when in actual contact with the mouth. Not only this, which is the least cause for alarm, but the constant fear that the saliva will force its way into the cavity, thereby rendering the filling imperfect, it seems to me is sufficient to convince any operator that he ought to use the appliance which will most successfully exclude the moisture during the operation.

Some dentists claim, should the filling get wet, they can cut away a portion of the gold and proceed with the operation. Why bring about such conditions? Why not apply the rubber dam in the first place, save time, and avoid all such danger? So far as trouble and loss of time is concerned, we believe it may all be attributed to a lack of knowledge as to how to adjust this appliance. But few cases should require more than five, and rarely over two or three minutes for this operation. To adjust it on the anterior teeth. First, to render the work easier, where the teeth are crowded, draw a piece of floss silk, well waxed, between them. After making the required number of holes, stretch the dam sufficiently tight to enable you to gradually force it between the teeth. If it cannot be done in this way, place the spaces in the dam as far as possible between the teeth, and complete the operation by forcing it down with floss silk. Using floss silk, place a thread around the crown of each tooth enveloped, grasping the ends firmly with one hand, and with a flat burnisher gradually force the dam to its proper position, around the necks of the teeth. After which, if necessary to hold the dam in position, or more perfectly exclude the moisture, secure it with a thread around only those teeth necessary to facilitate the operation. In a great many cases this will be unnecessary, as the tension of the dam, aided by the peculiar shape of the teeth, will be sufficient to hold it in its proper position. In other, and in fact, in the majority of cases, it will be necessary to secure it only on the tooth, or teeth operated upon; hence avoiding the little pain—the only one of any consequence caused by the use of the dam. To adjust the dam over the molars, and sometimes the bicuspids: After making the required number of holes; in which you will be governed by the tooth, or teeth to be operated upon, and the location of the cavity; if in the crown, only two, and many times but one is necessary; if on the buccal, or lingual surface the same rule will apply; if on the proximal surface it will be necessary to place it over two, sometimes three, and in many cases four teeth in order to keep the dam out of the way of the operator and give free access to the cavity. Stretch the dam over that portion of the clamp which is to envelope the tooth; then, with the clamp on the forceps (clamp-forceps), the dam folded around them, place the clamp on the last tooth back to be operated upon. If the cavity is posterior proximal, on the tooth immediately back of it, holding the loose dam with one hand, with a flat burnisher—or the fingers—gradually draw it forward till it passes underneath the clamp and envelops the tooth. The remaining work is easily completed by drawing the dam forward over each succeeding tooth and, when necessary, securing it around the teeth with floss silk; after which the upper part of the dam should be held in position, and out of the way of the operator, by the rubber dam holders.

When the operator has no clamps convenient, place the dam first over the anterior tooth, on which it is to be applied, securing it with a thread; then over each succeeding tooth back till the operation is complete.

The objection is sometimes made that the cavity extends too far under the margin of the gum to apply the dam. To an experienced operator but few such cases will appear, as he can readily find clamps of such shape and strength as will force the gum out of the way, and even under it beyond the margin of the cavity, or to such an extent that in nearly all cases it will exclude more moisture at the point of exposure than any other method. While it not only excludes the moisture, it gives the operator free use of both hands, which is necessary in rapid operating and perfect work.

Some of the non-rubberdamists say that their patients express themselves as being so glad they do not use the rubber dam, as it is so annoying. But do they explain to their patient that their competitor who used the dam did it because he could do his work easier, and perform a more perfect and, to himself and the patient, satisfactory operation? We fear not. Again, they do not know how many patients go to the dentist of rubber dam fame and say they prefer the use of the dam for various reasons. Only a few days since I had a patient, who had never known the use of this appliance, say she thought it a great improvement for various reasons. Gave as one advantage, "It keeps the operator and patient from breathing in each other's mouth." This statement we can all readily appreciate, as, not only some dentists, but a great many patients, have a breath, the odor of which will pass through a brick wall and cause a man to faint on the opposite side.—*London Dental Record.*

